# Clinical efficacy of two modalities of selective laser trabeculoplasty in the treatment of primary open-angle glaucoma

**DOI:** 10.1177/11206721251372801

**Published:** 2025-09-17

**Authors:** Abdelrahman Assaf, Fidan A. Aghayeva, Tatiana Sykorova, Ines Lanzl

**Affiliations:** 1 Department of Ophthalmology, University of Ulm, Ulm, Germany; 2 Chiemsee Eye Clinic, Prien on Chiemsee, Prien am Chiemsee, Germany; 3 Technical University of Munich, Munich, Germany

**Keywords:** Glaucoma, intraocular pressure, intraocular pressure-lowering medications, selective laser trabeculoplasty

## Abstract

**Purpose:**

To evaluate clinical outcomes of two selective laser trabeculoplasty (SLT) approaches in the management of primary open-angle glaucoma (POAG): a single-session 180° SLT versus a staggered 360° SLT approach, where a second 180° treatment was added to the superior trabecular meshwork 1–2 months after the initial inferior treatment.

**Methods:**

A retrospective chart review was conducted on 134 eyes of 134 patients with POAG treated at a single center. Sixty-seven eyes received only 180° SLT, while 67 underwent staggered 360° SLT. The decision to proceed with the second session was based on clinician judgment and was not standardized. Intraocular pressure (IOP) and the number of topical IOP-lowering medications were assessed at baseline, and at 3, 6, and 12 months post-treatment.

**Results:**

Both groups showed significant IOP reduction at all follow-up visits (p < 0.001). At 12 months, the mean IOP change was −5.1 ± 3.3 mmHg (180° SLT) and −4.9 ± 3.5 mmHg (360° SLT). Higher baseline IOP was associated with greater IOP reduction (p < 0.001). No statistically significant difference in IOP-lowering efficacy was observed between groups. However, the 360° SLT group showed a greater within-group reduction in medication burden (p < 0.01) compared to the 180° group (p < 0.05).

**Conclusions:**

Both 180° and staggered 360° SLT were effective in lowering IOP in POAG patients. While limited by its retrospective design and selection bias, this study supports staggered SLT as a viable strategy to reduce medication dependency in clinical practice.

## Key messages

Selective Laser Trabeculoplasty (SLT) is a well-established, safe, and cost-effective first-line treatment for ocular hypertension and open-angle glaucoma. In our real-world cohort, 180° and staggered 360° SLT demonstrated comparable intraocular pressure (IOP)–lowering efficacy over 12 months. However, 360° SLT was associated with a greater reduction in the need for topical IOP-lowering medications, resulting in a higher proportion of drop-free patients. This underscores the potential advantage of a staggered 360° approach in reducing medication burden, even when the IOP reduction itself is similar.

## Introduction

Open-angle glaucoma (OAG) is the most common form of glaucoma, with a prevalence of approximately 2.4% in adults over 40 years of age,^1^ and its main development risk factor is elevated intraocular pressure (IOP). Topical IOP-lowering drops were considered to be the standard primary treatment of glaucoma during a long time period until new modalities of laser treatment had been developed.

Selective laser trabeculoplasty (SLT) was introduced in 1995 by Latina and Park and received United States Food and Drug Administration approval in 2001. Since then SLT has been increasingly used as a primary or an adjunct treatment in outpatient-based clinical practice.^2-5^ The effect of SLT is based on a process known as selective photothermolysis with using a532 nm Q-switched, frequency-doubled Nd:YAG laser that delivers a shorter pulse duration (3 ns) compared to ALT (∼0.1 s) and prevents heat dissipation outside of pigmented trabecular meshwork (TM) cells, thus is less associated with the main adverse events related to ALT, such as a peripheral anterior synechiae development, a transient acute IOP rise following laser, corneal endothelial changes, and acute anterior uveitis.^2,3,6-8^ This laser treatment option has also been proposed to reduce diurnal IOP instability, and thus, prevent glaucoma progression,^2,3,9,10^ furthermore it can be repeated with similar effectiveness to the primary procedure.^11,12^ This procedure is supposed to be a promising option to provide freedom from eye drops, especially for non compliant patients. Different clinical trials comparing SLT with other glaucoma treatment options, including medical therapy, ALT and surgery have been conducted. In those different modalities of SLT (90°, 180° and 360° SLT) were performed.^2,13-19^ However, until recently, it was a controversial discussion in the literature whether the effect of SLT is not largely temporary; whether SLT is clinically effective enough as a first-line treatment, and as a monotherapy to achieve a given target IOP in patients with glaucoma and ocular hypertension (OHT). After the publication of the 3-year results of the LIGHT trial, the American Academy of Ophthalmology, the European Glaucoma Society, and the National Institute for Health and Care Excellence updated their glaucoma management guidelines on glaucoma treatment, and SLT was listed as initial or as a recommended first-line treatment for OAG and OHT alongside medications.^20-26^

The aim of this study is not to directly compare treatment efficacy, but rather to evaluate the clinical outcomes of two SLT treatment approaches for primary open-angle glaucoma (POAG) in a community setting: (1) a single-session 180° SLT delivered to the inferior trabecular meshwork, and (2) a staggered 360° SLT approach, in which the inferior 180° is treated initially and the superior 180° is treated 1–2 months later, as deemed appropriate by the treating physician.

## Material and methods

We performed a retrospective chart review of 134 patients (134 eyes) with POAG who underwent SLT treatment at Chiemsee Eye Clinic, Germany. 67 patients (67 eyes) underwent 180° SLT, while 67 patients (67 eyes) received a staggered 360° SLT protocol, in which a second 180° session was performed based on clinical judgment. The study adhered to the tenets of the Declaration of Helsinki and informed consent was obtained from all the patients.

We used National Institute for Health and Clinical Excellence (NICE) thresholds for disease definition (OAG or OHT) and treatment initiation. According to NICE patients have the right to be able to make informed decisions about their care. To diagnose chronic open angle glaucoma and related conditions it is recommended to offer all of the following tests: visual field assessment using standard automated perimetry; optic nerve assessment and fundus examination using stereoscopic slit lamp biomicroscopy, with pupil dilatation; IOP measurement using Goldmann applanation tonometry (slit lamp mounted); peripheral anterior chamber configuration and depth assessments using gonioscopy; and central corneal thickness (CCT) measurement.^25^ Analysis of the optic disc and CCT measurement in our study were done using Optical coherence tomography (SD OCT Revo, Optopol), visual filed damage was determined by automated visual field examination using the Medmont M700 Automated Perimeter (MAP, Australia), IOP measurements were obtained with Goldmann applanation tonometry, and gonioscopy was performed by using a 4-mirror Sussmann Goniolens. Patients with only one functional eye (monocular patients) or patients who underwent any previous intraocular surgery with exclusion of cataract surgery, performed in the time period at least one year before indication of SLT, were excluded from the study. We included only one eye (right eye) per patient from those who underwent SLT procedure for both eyes. According to NICE treatment recommendation for people with chronic OAG 360° SLT was initially offered to all our included glaucoma patients (primary diagnosed and already treated) to delay the need for additional eye drops to achieve the target IOP or because of the adverse events with intolerance of IOP-lowering drops and incompliance. 360° SLT was planned as two successive 180° SLT procedures with the time interval 4 weeks. Only one 180° SLT procedure was performed for patients who achieved target IOP after the first procedure or did not want to undergo a second 180° SLT procedure and preferred additional glaucoma medications. This introduces a degree of selection bias, which limits direct comparisons between the groups. Eye-specific target IOP was determined by NICE and European Glaucoma Society guidance according to stage of disease/visual field damage at diagnosis, the baseline IOP and disease progression rate. IOP level of 18 to 20 mmHg with a reduction of at least 20% from baseline IOP, 15 to 17 mmHg with a reduction of at least 30% from baseline IOP, and 10 to 12 mmHg was defined as a target IOP in early, in moderate and in advanced glaucoma, respectively.^21,25,26^

Post-SLT medications were to include Difen UD 1 mg/mL (Diclofenac natrium 0.1%) eye drops four times daily during 5 days and moisturizing eye drops. Main outcome measures in this study were pre- and post-SLT IOP levels and IOP change after SLT. Secondary outcome was the number of topical IOP-lowering medications during follow-up after SLT. Pre-SLT IOP was taken at the time of SLT indication. The amount and percentage of IOP change from pre-SLT level in the treated eye was evaluated at 3, 6 and 12 months follow up after SLT.

### Selective laser trabeculoplasty procedure

SLT was performed using topical anaesthetic drops and a gonioscopic lens (Ocular Latina SLT Gonio Laser), with coupling medium. Laser treatment was delivered at 180° or 360° of the trabecular meshwork with 50–60 or 100–120 nonoverlapping shots (the spot size 400 microns, 25 per quadrant; energy, 0.3 to 1.5 mJ) and with a just visible tissue reaction or small microbubbles.

### Statistical analysis

All statistical analysis was conducted with IBM^®^ SPSS^®^ Statistics (Statistical Package for the Social Sciences). Absolute and relative frequencies were computed for dichotomous data, continuous data are presented as mean ± standard deviation(range). Paired Wilcoxon test was used to compare pre-SLT and post-SLT IOP, and pre- and post-SLT number of medications in both groups. Correlations between the IOP change from baseline to follow-up in the treated eye was analyzed using Spearman's rank correlation. Descriptive data was compared using Chi-Square and Mann-Whitney U tests.

## Results

The mean age of all patients was 70.4 ± 10.8 years; 74 (55.2%) women and 60 (44.8%) men. 38.1% eyes were pseudophakic, while 61.9% were naïve to any previous intraocular surgery. The demographic and clinical data of all patients in both groups is presented in Table 1.

**Table 1.** Demographic and clinical data of all patients.

Data	180° SLT group	360° SLT group
Number of patients/eyes	67/67	67/67
Mean age ± SD (range)*, years	69.3 ± 10 (47–95)	71.5 ± 11.6 (49–93)
Sex*, female / male	42 (62.7%) / 25 (37.3%)	32 (47.8%) / 35 (52.2%)
Mean duration of glaucoma*, years	7.6 ± 6.5	6.8 ± 6.6
Severity of glaucoma*		
mild	27 (40.3%)	28 (41.8%)
moderate	32 (47.8%)	30 (44.8%)
advanced	8 (11.9%)	9 (13.4%)
Lens status		
phakic	47 (70.1%)	36 (53.7%)
pseudophakic	20 (29.9%)	31 (46.3%)
IOP_max_ (mmHg)*	27.5 ± 8.8	28.6 ± 8.5
CCT (µm)*	537.9 ± 40.5	534.6 ± 34.9
MD (dB)*	–4.3 ± 3.2	–4.4 ± 4.1

SLT = selective laser trabeculopasty; SD = standard deviation; IOP_max_ = maximum baseline intraocular pressure; CCT = central corneal thickness; MD = mean deviation (Medmont perimetry).

* no statistically significant differences in study parameters between groups.

**Figure 1.**
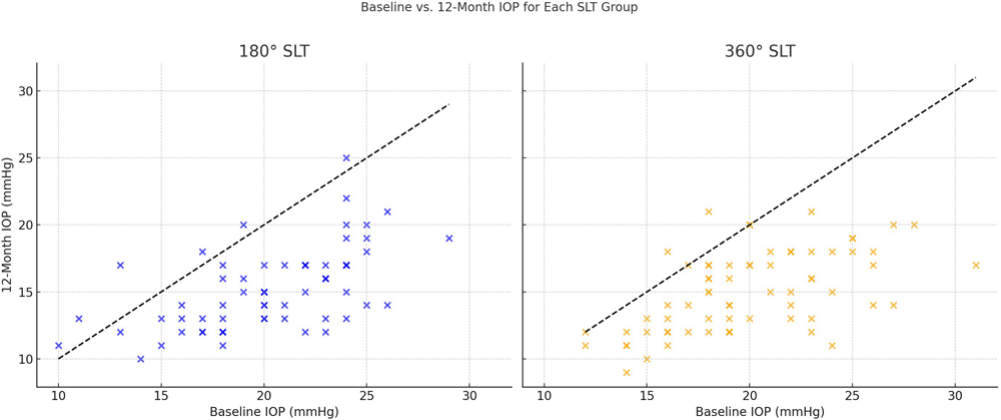
Intraocular pressure (IOP) at baseline vs. 12-Month Follow-Up in SLT-Treated Eyes.

The mean pre-180° SLT and post-180° SLT IOP in the treated eye at 3, 6 and 12 months were 20.4 ± 3.9 (10–29) mmHg, 15.7 ± 2.8 (9–22) mmHg, 16.2 ± 3 (12–31) mmHg and 15.2 ± 3 (10–25) mmHg, respectively. The mean pre-360° SLT and post-360° SLT IOP in the treated eye at 3, 6 and 12 months were 19.9 ± 4.1 (12–31) mmHg, 14.8 ± 3.3 (7–25) mmHg, 15.2 ± 3.8 (8–32) mmHg and 15 ± 3 (9–21) mmHg, respectively. The pre-SLT IOP was not significantly different between two groups. The mean post-SLT IOP levels were statistically significantly lower than pre-SLT IOP levels at all follow-ups after 180° SLT and 360° SLT (–6.47 and −6.69, *p* < 0.001; −5.69 and −6.4, *p* < 0.001; −6.69 and −6.74, *p* < 0.001 at 3, 6 and 12 months, respectively) Figure 1.

Left: 180° SLT group–each dot represents an individual patient's IOP at baseline (x-axis) and at 12 months (y-axis). The dashed identity line indicates no change; points below the line represent IOP reduction. Right: 360° SLT group–same representation for patients treated with the staggered 360° SLT protocol (inferior 180° followed by superior 180° after 1–2 months).

The mean IOP change in the treated eyes at 3, 6 and 12 months after 180° SLT was – 4.5 ± 3.1 (–13.0–5.0) mmHg, −4.1 ± 3.9 (–11.0–7.0) mmHg, and - 5.1 ± 3.3 (–12.0–4.0) mmHg, respectively. The mean IOP change in the treated eyes at 3, 6 and 12 months after 360° SLT was - 5.1 ± 3.5 (–16.0–0) mmHg, - 4.8 ± 3.6 (–15.0–6) mmHg, and - 4.9 ± 3.5 (–14.0–3.0) mmHg, respectively. Thus, IOP reduction at 3-, 6- and 12-months follow-up after 180° and 360° SLT was 22.1% and 25.6%; 20.1% and 24.1%; 25% and 24.6%, respectively.

We have not found any statistically significant difference between IOP reduction after 180° SLT and 360° SLT during the follow-up, while 360° SLT led to a greater level of statistically significant reduction in the mean number of pre-SLT medications at 6 months (*p* < 0.001 vs. *p* < 0.05) and at 12 months (*p* < 0.01 vs. *p* < 0.05) after laser treatment in compare to 180° SLT. However, comparison of IOP-lowering medication number between the groups did not reveal any significant difference both pre-SLT and at different follow-ups post-SLT. In patients without achieved target IOP level after 180° SLT or 360° SLT additional IOP-lowering medications were prescribed. 29.9% of patients after 360° SLT vs. 24.2% of patients after 180° SLT needed no IOP-lowering medications at 12 months follow-up. Target IOP was achieved under glaucoma monotherapy in 54.5% of patients after 180° SLT and in 41.8% of patients after 360° SLT. The mean number of IOP-lowering medications and percentage distribution of the eyes with different number of medications pre-SLT and at different follow-ups post-SLT in both groups are presented in Table 2.

**Table 2.** Mean number of IOP-lowering medications and distribution of the eyes (%) with different number of medications pre-SLT and at different follow-ups post-SLT in both groups.

Number	Pre-SLT		3 months post-SLT		6 months post-SLT		12 months post-SLT	
	180° SLT	SLT 360°	180° SLT	SLT 360°	180° SLT	SLT 360°	180° SLT	SLT 360°
Mean ±SD	1.21 ± 0.80	1.42 ± 1.0	0.95 ± 0.7**	1.34 ± 1.2	1.0 ± 0.7*	1.06 ± 0.9***	1.0 ± 0.7*	1.12 ± 1.0**
P-Value	0.5		0.1		1.0		0.8	
0	16.4	6.0	24.2	26.2	22.7	30.2	24.2	29.9
1	53.7	70.1	57.6	36.9	56.1	42.9	54.5	41.8
2	22.4	10.4	16.7	21.5	19.7	19.0	18.2	16.4
3	7.5	3.0	1.5	7.7	1.5	6.3	3.0	10.4
4	–	10.4	–	7.7	–	1.6	–	1.5

IOP = intraocular pressure; SLT = selective laser trabeculopasty; SD = standard deviation.

Mann-Whitney Test; P-value: no statistically significant difference between 180° SLT and 360° groups.

Wilcoxon Signed Ranks Test: statistically significant difference between pre-SLT and post-SLT mean parameters in 180**°** SLT and 360**°** SLT groups; *p < 0.05; **p < 0.01; ***p < 0.001

In the staggered 360° SLT group, the proportion of patients using no topical glaucoma medications increased from 4% at baseline to 20% at 12 months post-treatment. In the 180° SLT group, this proportion rose from 11% to 16% over the same period. However, this difference was not statistically significant (***p*** **=** **0.56**).

At baseline, the mean number of IOP-lowering medications was 1.21 ± 0.80 in the 180° SLT group and 1.42 ± 1.00 in the staggered 360° SLT group, with no statistically significant difference between them (p = 0.5).

During the 12-month follow-up period, both groups exhibited a reduction in medication use, although no statistically significant differences were observed between them at any time point. At 3 months, the mean number of medications was 0.95 ± 0.70 in the 180° group and 1.34 ± 1.20 in the 360° group (p = 0.1). At 6 months, it was 1.00 ± 0.70 in the 180° group compared to 1.06 ± 0.90 in the 360° group (p = 1.0). By 12 months, the mean remained stable at 1.00 ± 0.70 in the 180° group and was 1.12 ± 1.00 in the 360° group (p = 0.8).

These findings indicate that while medication use decreased over time in both treatment groups, there were no statistically significant differences in medication burden between the two approaches at any follow-up interval.

No complications were mentioned during SLT procedure in both groups. Mild anterior chamber inflammation and transient IOP elevation in the first week after SLT were noted in 5 (3.7%) patients (3% in 180° SLT group vs. 4.5% in 360° SLT group) and in 7 (5.2%) patients (4.5% in 180° SLT group vs. 6% in 360° SLT group) transient macular oedema with resolution during four weeks under Nevanac (Nepafenac 0.1%) drops was mentioned in one patient after 360° SLT.

Comparison of the overall complication rate between 180° SLT and 360° SLT groups did not reveal any significant difference (*p* = 0.38).

## Discussion

According to the literature review SLT has been predicted to be a safe, clinically and cost-effective procedure, and is currently offered as a first-line treatment for open angle glaucoma and ocular hypertension.^27-29^ The largest prospective evaluation to date of the SLT effectiveness, the Laser in Glaucoma and Ocular Hypertension (LiGHT) Trial is a multicenter randomized controlled trial comparing initial treatment with SLT with initial treatment with IOP-lowering eye drops for 718 treatment-naïve patients with OAG or OHT and showed that 74.2% of patients after SLT required no drops and no glaucoma surgery to maintain target IOP versus 11 (1,8%) patients operated in eye drops group with 97% probability of SLT as first treatment being more cost-effective than topical treatment at 3 years follow up.^22-24^

Several studies compared different modalities of SLT, and reported results are controversial. Goyal et al. did not find significant differences between 360° SLT and 180° SLT groups in terms of the increase in tonographic outflow facility and IOP reduction.^15^ Shibata et al. demonstrated that IOP reduction rate in 69 Japanese eyes with OAG after 360° SLT stayed statistically higher than after 180° SLT.^14^ Another study suggests that 360° SLT is associated with smaller IOP fluctuations than 180° SLT and therefore is more efficacious.^16^ Two meta-analysis included nine studies, involving 1044 and 1065 eyes, were performed by Zhu et al and by Amaral et al. and showed that 360° SLT demonstrated superior IOP reduction in compare to 180° SLT at 12 months.^17,18^ However, in a RCT performed by Nagar et al. no significant difference was found in terms of success rates between 360° SLT and 180° SLT groups.^19^

Our study demonstrates that both 180° SLT and 360° SLT procedures lead to a significant IOP reduction from baseline at all follow-ups after SLT *(p* *<* *0.001*), the between-group comparison did not yield a significant difference (*p* = 0.4) during the follow-up, and these results confirm the data of several previously performed clinical trials.^13,15,19^ While the study may be underpowered to detect very small differences between groups, the lack of a significant difference does not imply equivalence but rather reflects the clinical reality that both treatment approaches appear similarly effective within the observed range. SLT has been proven to be a clinically effective treatment option, as a first line treatment in 11.2% and as an adjunct treatment in 88.8% of all our cases.

Average IOP reduction following SLT is reported to be 21.8–29.4% at 6 months and 16.9–30% at 12 months; success rates vary from 66.7 to 75% eyes at 6 months and 58 to 94% at 12 months.^2,30^ In our study IOP reduction was 20.1% and 24.1% at 6 months, and 25% and 24.6% at 12-months follow-up after 180° and 360° SLT, respectively. According to previously published data no correlations were found between higher energy level or laser pulse duration during SLT and IOP-lowering effect.^31,32^

A larger IOP-lowering effect after SLT at different follow-ups in our study was associated with a higher pre-SLT IOP in the treated eye (*p* < 0.001). Elevated baseline IOP was reported to be the success predictor of SLT previously.^33^ Furthermore, Pillunat et al. suggested that SLT has no success rates in patients with pretreatment baseline IOP <14 mm Hg.^34^ McIlraith et al. reported that IOP reduction in eyes on topical IOP-lowering drops discontinued 1 month before SLT was significantly less compared to the treatment-naive group after laser treatment and these results are controversial to some other published data.^35,36^ 88.8% patients in our study were already on topical glaucoma medications at the time of SLT indication. There was not any statistically significant difference in the mean number of IOP-lowering medications at different follow-ups post-SLT between groups, while 360° SLT was slightly more effective than 180° SLT in terms of reducing the number of IOP-lowering medications needed at 6 (*p* < 0.001 vs. *p* < 0.05) and 12 months (*p* < 0.01 vs. *p* < 0.05) follow-up. 29.9% of patients after 360° SLT (6% pre-SLT) vs. 24.2% of patients after 180° SLT (16.4% pre-SLT) needed no IOP-lowering medications at 12 months follow-up. Thus, the number of drops free patients increases up to 7.8% and 23.9% while maintaining target IOP at 1 year after 180° SLT and 360° SLT, respectively. The number of glaucoma patients on 4-fold IOP-lowering therapy pre-SLT decreases from 10.4% to 1.5% at 12 months after 360° SLT. While the difference in the proportion of patients who were medication-free at 12 months between the 180° SLT group (24.2%) and the staggered 360° SLT group (29.9%) was not statistically significant (p = 0.56), the absolute increase from baseline is notable. In the 180° SLT group, the percentage of drop-free patients rose from 16.4% to 24.2%, and in the 360° SLT group, from 6.0% to 29.9%. These shifts represent meaningful clinical improvements in both groups. The lack of statistical significance may be attributed to the limited sample size, which reduces the power to detect moderate between-group differences. Nevertheless, the findings underscore the potential of SLT—especially when applied in a staged manner—to reduce the dependency on topical IOP-lowering medications, which is particularly valuable in populations with adherence challenges or medication-related side effects.

Among SLT-related ocular adverse events noted in 6% of the patients the following was reported: inflammation after SLT in 0.3%, rise in IOP after SLT in 1.0%-1.7%, development of peripheral anterior synechiae in 2.9%, other transient events like discomfort, blurred vision, photophobia, and hyperemia in 24.6%-34.4%, adverse events during procedure in 2%.^23,24,29^ According to other published results IOP rise of ≥ 5 mmHg and anterior chamber inflammation could occur in up to 28% and 83% of eyes.^33,37^ According to performed meta-analysis included nine studies comparing 180° SLT and 360° SLT, adverse event rates did not significantly differ between the groups.^17,18^ No significant differences in the incidence of postoperative IOP spikes after both modalities of SLT were reported by Nirappel et al..^38^

In our study no adverse events during SLT procedure itself were registered; transient adverse events post-SLT included anterior chamber inflammation in 3.7%, IOP elevation in 5.2% of all cases and transient macular edema in one patient. The overall rate of post-SLT complications consisted 7.5% in 180° SLT and 11.9% in 360° SLT groups. Moreover, Chi-Square Test revealed no significant difference in complication rate per complication between the two groups, and Mann-Whitney U Test showed no significant difference in overall complication rate between 180° and 360° SLT groups (*p* = 0.38). We found no sight-threatening complications of SLT, such as corneal changes (corneal edema and stromal scarring), retinal changes (persistent cystoid macular edema, development of subretinal fluid) or choroidal effusions.^37,39^

The published data regarding topical anti-inflammatory drops post SLT remains controversial, so neither a prospective RCT evaluating the use of topical anti-inflammatory drops (indomethacin 0.1% or dexamethasone 0.1%) for one-week vs control nor several other studies found evidence and a statistically significant difference in the grade of inflammatory response between groups.^40,41^ In contrast, Groth et al in their SALT trial reported significantly better IOP reduction at 3 months in eyes treated with topical steroids or NSAID drops after SLT.^42^ Our patients have used prescribed Difen (Diclofenac natrium 0.1%) eye drops four times daily during 5 days.

One of the strengths of our community setting based study is that we included both glaucoma treatment naïve eyes and eyes already on topical glaucoma treatment. We included one eye per patient to avoid bias of the results and possible SLT dependent systemic effects like a consensual ophthalmotonic reaction.^43^ The main limitations are its retrospective design and the short follow-up. Thus, this study shows a statistically significant IOP reduction at 3, 6 and 12 months after both modalities of SLT. In our cohort, the decision to perform a second 180° SLT was made by the treating physician based on individualized clinical assessment and was not guided by a standardized IOP cutoff. SLT in our set up has proved to be a clinically effective treatment option and can be definitely used as a potential alternative primary or an adjunct treatment in patients with OAG and OHT in an outpatient-based clinical practice, especially in old or complicated patients with poor compliance and with comorbidity.

While the staggered 360° SLT approach may offer clinical benefits in selected cases, it inherently involves two separate treatment sessions instead of one. This has implications not only for patient convenience and clinic logistics, but also for healthcare expenditures—whether borne by the patient, national health systems, or insurance providers. The added session increases use of clinical time, laser equipment, and personnel resources. From the patient's perspective, multiple visits may pose barriers to adherence, especially in community-based or resource-limited settings.

At the same time, it remains unclear how many times SLT can be effectively repeated over the course of glaucoma management. In this context, an initial 180° treatment may offer a strategic advantage by preserving trabecular meshwork for future interventions. If partial treatment achieves sufficient pressure control or allows more frequent re-treatments over time, it may ultimately prove more cost-effective and clinically sustainable. Future studies should therefore assess not only the immediate efficacy, but also the long-term treatment burden and repeatability of 180° versus 360° SLT protocols to obtain optimal resource utilization.

Care should be taken to ensure regular follow up in patients who do not need pressure lowering eye drops after SLT, in order not to miss any future IOP increases in the development of this chronic disease, since disease awareness in patients without continuous eye drop therapy might be waning over time.

## Conclusion

In summary, our study reinforces the effectiveness and safety of both 180° and 360° SLT as valuable treatment modalities for patients with open-angle glaucoma. While 360° SLT showed a tendency towards a greater reduction in IOP-lowering medication use, no statistically significant differences in IOP reduction or complication rates were observed between the two approaches over a 12-month follow-up period. The choice between a single-session 360° SLT or a staggered 180° SLT should therefore be individualized, balancing clinical efficacy, patient adherence, and healthcare resource considerations. Given the chronic and progressive nature of glaucoma, long-term studies are warranted to evaluate the durability of SLT outcomes and to determine optimal retreatment strategies that maximize efficacy while minimizing treatment burden.

## References

[1] Zhang N Wang J Li Y , et al. Prevalence of primary open angle glaucoma in the last 20 years: a meta-analysis and systematic review. Sci Rep 2021; 11: 13762. 34215769 10.1038/s41598-021-92971-w PMC8253788

[2] Garg A Gazzard G . Selective laser trabeculoplasty: past, present, and future. Eye 2018; 32: 863–876. 29303146 10.1038/eye.2017.273 PMC5944654

[3] Garg A Vickersta V Nathwani N , et al. Primary selective laser trabeculoplasty for open-angle glaucoma and ocular hypertension: clinical outcomes, predictors of success, and safety from the laser in glaucoma and ocular hypertension trial. Ophthalmology 2019; 126: 1238–1248. 31028768 10.1016/j.ophtha.2019.04.012

[4] Latina MA Park C . Selective targeting of trabecular meshwork cells: in vitro studies of pulsed and cw laser interactions. Exp Eye Res 1995; 60: 359–371. 7789416 10.1016/s0014-4835(05)80093-4

[5] Realini T Shillingford-Ricketts H Burt D , et al. Long-term outcomes of selective laser trabeculoplasty for open-angle glaucoma in the Caribbean. Am J Ophthalmol 2021; 232: 83–89. 34153267 10.1016/j.ajo.2021.06.012 PMC8634933

[6] Anderson RR Parrish JA . Selective photothermolysis: precise microsurgery by selective absorption of pulsed radiation. Science 1983; 220: 524–527. 6836297 10.1126/science.6836297

[7] Kagan DB Gorfinkel NS Hutnik CM . Mechanisms of selective laser trabeculoplasty: a review. Clin Experiment Ophthalmol 2014; 42: 675–681. 24330092 10.1111/ceo.12281

[8] Wise JB Witter SL . Argon laser therapy for open-angle glaucoma: a pilot study. Arch Ophthalmol 1979; 97: 319–322. 575877 10.1001/archopht.1979.01020010165017

[9] Kiddee W Atthavuttisilp S . The effects of selective laser trabeculoplasty and travoprost on circadian intraocular pressure fluctuations: a randomized clinical trial. Medicine (Baltimore) 2017; 96: e6047. 10.1097/MD.0000000000006047 PMC5313007 28178150

[10] Nagar M Luhishi E Shah N . Intraocular pressure control and fluctuation: the effect of treatment with selective laser trabeculoplasty. Br J Ophthalmol 2009; 93: 497–501. 19106150 10.1136/bjo.2008.148510

[11] Ayala M Chen E . Comparison of selective laser trabeculoplasty (SLT) in primary open angle glaucoma and pseudoexfoliation glaucoma. Clinical Ophthalmology 2011; 5: 1469–1473. 22069348 10.2147/OPTH.S25636 PMC3206117

[12] Francis BA Loewen N Hong B , et al. Repeatability of selective laser trabeculoplasty for open-angle glaucoma. BMC Ophthalmol 2016; 16: 1–7. 27464887 10.1186/s12886-016-0299-9 PMC4964282

[13] McAlinden C . Selective laser trabeculoplasty (SLT) vs other treatment modalities for glaucoma: systematic review. Eye 2014; 28: 249–258. 24310236 10.1038/eye.2013.267 PMC3965810

[14] Shibata M Sugiyama T Ishida O , et al. Clinical results of selective laser trabeculoplasty in open-angle glaucoma in Japanese eyes: comparison of 180 degree with 360 degree SLT. J Glaucoma 2012; 21: 17–21. 21173708 10.1097/IJG.0b013e3181fc8020

[15] Goyal S Beltran-Agullo L Rashid S , et al. Effect of primary selective laser trabeculoplasty on tonographic outflow facility: a randomised clinical trial. Br J Ophthalmol 2010; 94: 1443–1447. 20472748 10.1136/bjo.2009.176024

[16] Prasad N Murthy S Dagianis JJ , et al. A comparison of the intervisit intraocular pressure fluctuation after 180 and 360 degrees of selective laser trabeculoplasty (SLT) as a primary therapy in primary open angle glaucoma and ocular hypertension. J Glaucoma 2009; 18: 157–160. 19225355 10.1097/IJG.0b013e3181752c97

[17] Zhu D Shah PP Wong A , et al. 180- Versus 360-degree selective Laser trabeculoplasty in open angle glaucoma and ocular hypertension: a systematic review and meta-analysis. J Glaucoma 2024; 33: 566–575. 38709197 10.1097/IJG.0000000000002415

[18] Amaral DC Guedes J Moreira PHS , et al. A comparison of the 360° versus 180° of selective Laser trabeculoplasty (SLT) in the treatment of open angle glaucoma (OAG) and ocular hypertension (OHT): a comprehensive systematic review and meta-analysis. Curr Eye Res 2025; 12: 1–11. 10.1080/02713683.2025.2485223 40219934

[19] Nagar Ogunyomade O'Brart , et al. A randomised, prospective study comparing selective laser trabeculoplasty with latanoprost for the control of intraocular pressure in ocular hypertension and open angle glaucoma. Br J Ophthalmol 2005; 89: 1413–1417. 16234442 10.1136/bjo.2004.052795 PMC1772946

[20] American Academy of Ophthalmology . American academy of ophthalmology. primary open-angle glaucoma preferred practice pattern. https://www.aao.org/education/preferred-practice-pattern/primary-open-angle-glaucoma-ppp; 2020. Accessed on April 1, 2022.

[21] Meier-Gibbons F , T ¨ oteberg-Harms M. The 5th edition of the guidelines by the European glaucoma society–what has changed from 2014 to 2020. Klinische Monatsblätter für Augenheilkunde 2022; 239:458–459. 35472786 10.1055/a-1785-4481

[22] Gazzard G Konstantakopoulou E Garway-Heath D , et al. Laser in glaucoma and ocular hypertension (light) trial. a multicentre, randomised controlled trial: design and methodology. Br J Ophthalmol 2018; 102: 593–598. 28903966 10.1136/bjophthalmol-2017-310877

[23] Gazzard G Konstantakopoulou E Garway-Heath D , et al. Light trial study group. Selective laser trabeculoplasty versus eye drops for first-line treatment of ocular hypertension and glaucoma (LIGHT): a multicentre randomised controlled trial. Lancet 2019; 393: 1505–1516. 30862377 10.1016/S0140-6736(18)32213-X PMC6495367

[24] Gazzard G Konstantakopoulou E Garway-Heath D , et al. Laser in glaucoma and ocular hypertension (LIGHT) trial: six-year results of primary selective laser trabeculoplasty versus eye drops for the treatment of glaucoma and ocular hypertension. Ophthalmology 2023; 130: 139–151. 36122660 10.1016/j.ophtha.2022.09.009

[25] National Institute for Health and Clinical Excellence . NICE: guidance on glaucoma: diagnosis and management of chronic open angle glaucoma and ocular hypertension. DoH, URL: http://www.nice.org.uk/CG85fullguideline; 2017. Accessed January 27, 2022

[26] European glaucoma society terminology and guidelines for glaucoma, 5th edition. Br J Ophthalmol 2021; 105: 1–169. 10.1136/bjophthalmol-2021-egsguidelines 34675001

[27] Landers J . Selective laser trabeculoplasty: a review. Clin Experiment Ophthalmol 2021; 49: 1102–1110. 34331388 10.1111/ceo.13979

[28] Zhou R Sun Y Chen H , et al. Laser trabeculoplasty for open-angle glaucoma: a systematic review and network meta-analysis. Am J Ophthalmol 2021; 229: 301–313. 32888900 10.1016/j.ajo.2020.07.046

[29] Wong MOM Lee JWY Choy BNK , et al. Systematic review and meta-analysis on the efficacy of selective laser trabeculoplasty in open-angle glaucoma. Surv Ophthalmol 2015; 60: 36–50. 25113610 10.1016/j.survophthal.2014.06.006

[30] Weinand F Althen F . Long-term clinical results of selective laser trabeculoplasty in the treatment of primary open angle glaucoma. Eur J Ophthalmol 2006; 16: 100–104. 16496252 10.1177/112067210601600116

[31] Tang M Fu Y Fu MS , et al. The efficacy of low-energy selective laser trabeculoplasty. Ophthalmic Surgery, Lasers and Imaging Retina 2011; 42: 59–63. 10.3928/15428877-20101124-07 21117578

[32] Stunf Pukl S Drnovšek-Olup B . Impact of laser pulse duration on the reduction of intraocular pressure during selective laser trabeculoplasty. Int Ophthalmol 2018; 38: 83–91. 28040851 10.1007/s10792-016-0426-x

[33] Kennedy JB SooHoo JR Kahook MY , et al. Selective laser trabeculoplasty: an update. Asia-Pacific J Ophthalmol 2016; 5: 63–69. 10.1097/APO.0000000000000175 26886122

[34] Pillunat KR Spoerl E Elfes G , et al. Preoperative intraocular pressure as a predictor of selective laser trabeculoplasty ecacy. Acta Ophthalmol (Copenh) 2016; 94: 692–696. 10.1111/aos.13094 27213294

[35] Lee J Liu C Chan J , et al. Predictors of success in selective laser trabeculoplasty for primary open angle glaucoma in Chinese. Clin Ophthalmol 2014; 8: 1787–1791. 25228796 10.2147/OPTH.S69166 PMC4164283

[36] McIlraith I Strasfeld M Colev G , et al. Selective laser trabeculoplasty as initial and adjunctive treatment for open-angle glaucoma. J Glaucoma 2006; 15: 124–130. 16633226 10.1097/00061198-200604000-00009

[37] Song J . Complications of selective laser trabeculoplasty: a review. Clin Ophthalmol 2016; 10: 137–143. 26834456 10.2147/OPTH.S84996 PMC4716769

[38] Nirappel A Klug E Ye R , et al. Effectiveness of selective Laser trabeculoplasty applied to 360° vs. 180° of the angle. J Ophthalmol 2021; 2021: 8860601. 33643665 10.1155/2021/8860601 PMC7902141

[39] Knickelbein JE Singh A Flowers BE , et al. Acute corneal edema with subsequent thinning and hyperopic shift following selective laser trabeculoplasty. J Cataract & Refractive Surg 2014; 40: 1731–1735. 10.1016/j.jcrs.2014.08.002 PMC5518783 25263043

[40] Jinapriya D D’Souza M Hollands H , et al. Anti-inflammatory therapy after selective laser trabeculoplasty: a randomized, double-masked, placebo-controlled clinical trial. Ophthalmology 2014; 121: 2356–2361. 25234015 10.1016/j.ophtha.2014.07.017

[41] Realini T Charlton J Hettlinger M . The impact of anti-inflammatory therapy on intraocular pressure reduction following selective laser trabeculoplasty. Ophthal Surg, Lasers and Imag Retina 2010; 41: 100–103. 10.3928/15428877-20091230-18 20128578

[42] Groth SL Albeiruti E Nunez M , et al. SALT Trial: steroids after laser trabeculoplasty: impact of short-term anti-inflammatory treatment on selective laser trabeculoplasty efficacy. Ophthalmology 2019; 126: 1511–1516. 31444008 10.1016/j.ophtha.2019.05.032 PMC6810843

[43] Rhodes KM Weinstein R Saltzmann RM , et al. Intraocular pressure reduction in the untreated fellow eye after selective laser trabeculoplasty. Curr Med Res Opin 2009; 25: 787–796. 19203300 10.1185/03007990902728316

